# Construction of a prognostic risk-scoring model based on SASP-related genes in patients with skin cutaneous melanoma

**DOI:** 10.1007/s12672-025-02935-z

**Published:** 2025-08-27

**Authors:** Ming-Feng Li, Jing Du, Gang Wang, Wei Feng, Ju-Gao Chen, Chao Zhang

**Affiliations:** 1 Zhanjiang Institute of Clinical Medicine, Central People’s Hospital of Zhanjiang, Guangdong Medical University Zhanjiang Central Hospital, Zhanjiang, 524045 People’s Republic of China; 2 Department of Pathology, Central People’s Hospital of Zhanjiang, Guangdong Medical University Zhanjiang Central Hospital, Zhanjiang, 524045 People’s Republic of China; 3 Department of Oncology, Shenzhen People’s Hospital, Second Clinical Medical College of Jinan University, First Affiliated Hospital of Southern University of Science and Technology, Shenzhen, 518020 People’s Republic of China

**Keywords:** Senescence-associated secretory phenotype (SASP), Immune, Immune checkpoint, Tumor microenvironment (TME), Prognosis, Melanoma

## Abstract

**Background:**

Skin cutaneous melanoma (SKCM) is a highly aggressive and deadly subtype of skin cancer. Lack of efficient biomarkers for prognosis has limited the improvement of survival outcome for patients with SKCM.

**Methods:**

In this study, we obtained RNA-seq data from TCGA and GTEx databases, followed by identification of differential expressed genes, univariate Cox regression, and LASSO regression to identify prognostic SASP-related genes in the TCGA datasets and constructed a prognostic risk-scoring model.

**Results:**

The establishment of prognostic model was based on the expression levels of 14 SASP-related genes, including *ASPRV1*, *ICAM1*, *IL2RA*, *ABCC2*, *HLA-B*, *TPMT*, *ATM*, *CD59*, *KIR2DL4*, *CTLA4*, *ITGB3*, *FOXM1*, *NOX4*, and *TRIM21*. Patients with melanoma who were in the high-risk group had a shorter overall survival (OS), indicating that the model served as an independent prognostic index. Furthermore, we found that the risk score was potentially linked to immune scores, estimate score, immune cell infiltration level, and immunotherapy efficacy.

**Conclusions:**

This study presented a new prognostic model for assessing therapy in melanoma patients, providing a fresh perspective for combating melanoma.

## Introduction

Melanoma is a deadly malignancy that accounts for the majority of deaths in patients with skin cancer, despite comprising only 5% of skin cancers [1, 2]. During the past decade, the incidence of melanoma showed an upward trend worldwide [3]. Moreover, the occurrence and mortality rates of melanoma were also growing rapidly in China in recent years [4]. The prognosis of melanoma is related to various factors, such as clinical stage and response to systemic therapy. Although targeted therapy and immunotherapy have improved the survival outcome of patients with melanoma, they are not curative for these patients [5]. Therefore, there is an urgent need to identify effective and novel biomarkers for predicting treatment efficiency and prognosis in patients with melanoma.

Senescence has been revealed as an important tumor-suppressive mechanism in the early-stages of neoplastic transformation [6]. Senescent cells secrete numerous inflammatory cytokines, chemokines, and matrix proteases, which are termed as the senescence-associated secretory phenotype (SASP) [7]. Emerging studies have revealed that SASP contributes to attract immune cells that would help to eliminate senescent cells of tumor microenvironment (TME) [8]. Nevertheless, whatever its original purpose during senescence, the SASP can obviously have both advantageous and negative impacts on tumor. On one hand, SASP factors recruits immune cells to remove senescent cells and restore physiological function [9, 10]. On the other hand, weakened immunity induced by tumor also leads to a significant attenuate ability of SASP for subsequent scheduling immune system, thus accelerating the deterioration of TME [11]. Given the context-dependent function of SASP, comprehensively reconciling the differences in the tumor-suppressing and protumorigenic properties of the SASP are most compelling content in understanding the progression of tumor such as melanoma.

Herein, to identify biomarkers for risk stratification and accurate prognosis in patients with SKCM, we constructed a prognostic risk-scoring model based on the RNA expression levels of SASP-related genes and verified its performance in both of TCGA and GEO datasets. Furthermore, we investigated the associations between risk score and response to treatment of melanoma to evaluate the potential of the model for predicting treatment efficiency and prognosis in patients with melanoma.

## Materials and methods

### Data collection and processing

The RNA-seq data and clinical characteristics of TCGA-SKCM and GTEx datasets were downloaded from the University of California Santa Cruz Xena browser [12]. Patients with missing or ambiguous clinical information and follow-up data were excluded. The validation dataset was obtained from GEO database with an access number of GSE65904 [13]. The infiltration of immune cells and response to therapies were evaluated by using ESTIMATE algorithm [14]. Moreover, the response to immunotherapy was validated in 109 melanoma patients with pre- or on- immune checkpoint inhibitor (ICI) treatment from the GSE91061 dataset [15]. The list of SASP-related genes was acquired by searching with term of “senescence-associated secretory phenotype” in the GeneCards database [16].

### Construction and validation of the prognostic risk-scoring model

The R package DESeq2 [17] with a threshold of |log fold change (logFC)| ≥ 1 and *P* value < 0.05 was employed to identify SASP-related genes which were differentially expressed in tumor samples from TCGA-SKCM dataset as compared with normal samples from GTEx dataset. Meanwhile, the SASP-related genes significantly associated with length of overall survive (OS) time in patients from TCGA-SKCM dataset were identified by univariate Cox regression analyses with a threshold of *P* < 0.01. Subsequently, the intersected genes were selected for construction of the prognostic risk-scoring model by using LASSO Cox regression algorithm. Based on the coefficient of risk genes, we calculated the risk score for each SKCM patient according to the formula of ASPRV1 × 0.0853 + ICAM1 × (-0.0178) + IL2RA × (-0.0567) + ABCC2 × 0.0488 + HLA-B × (-0.0444) + TPMT × (-0.0960) + ATM × (-0.0784) + CD59 × (-0.0424) + KIR2DL4 × (-0.1577) + CTLA4 × (-0.0849) + ITGB3 × (-0.0026) + FOXM1 × 0.1364 + NOX4 × (-0.0619) + TRIM21 × (-0.0557). The same formula was used in both training and validation cohorts. Patients were assigned to high- or low-risk group according to the median cutoff value. Kaplan-Meier (K-M) curves and log-rank tests were applied to assess the difference in outcome of patients between the high- and low-low groups. The R package timeROC [18] was used to generate ROC curve and calculate area under time dependent ROC curve (AUC). Furthermore, a nomogram was constructed to visualize the relationship between variables and the prognostic model by the R package rms. The 1-year, 3-years, and 5-years calibration curves were applied to access the discrimination performance of model.

### Identification and functional annotation of the DEGs between high- and low-risk groups

The differentially expressed genes (DEGs) between high- and low-risk groups were identified by using R package DESeq2 with a threshold of |log fold change (logFC)| ≥ 1 and *P* value < 0.05. Furthermore, Kyoto Encyclopedia of Genes and Genomes (KEGG) pathway enrichment analysis and Gene Ontology (GO) functional annotation were carried out to explore the biological functions of DEGs between high- and low-risk groups by using R package clusterProfiler [19].

### Immune infiltration analysis and immunotherapy prediction

The fractions of 28 immune cell subtypes in each SKCM patient were assessed using single-sample gene set enrichment analysis (ssGSEA) with R package GSVA [20]. In addition, ESTIMATE algorithm [21] was used to compare the stromal score, immune score, ESTIMATE score, and tumor purity of patients between high- and low-risk groups. Moreover, target therapy and immune checkpoint inhibitors have been approved as routine drugs for the treatment of melanoma with remarkable success. Therefore, the expression levels of four immune checkpoints, PD-1(PDCD1), PD-L1(CD274), PD-L2(PDCD1LG2) and CTLA-4 included, and response to immunotherapy were compared in the high- and low-risk groups.

### Statistical analysis

Statistical analyses were carried out by using R (version 4.1.2). Survival curves were estimated using the Kaplan–Meier method. Overall survival was defined as time from the first treatment after primary diagnosis to death of any cause. As appropriate, the differences between the high- and low-risk groups were determined using two-tailed Chi-square test for a categorical variable and two-tailed Student’s t-test for a continuous variable. *P* < 0.05 was considered to indicate a significant difference, unless otherwise stated.

## Results

### Construction of prognostic risk-scoring model based on SASP-related genes in patients with melanoma

**Fig. 1:**
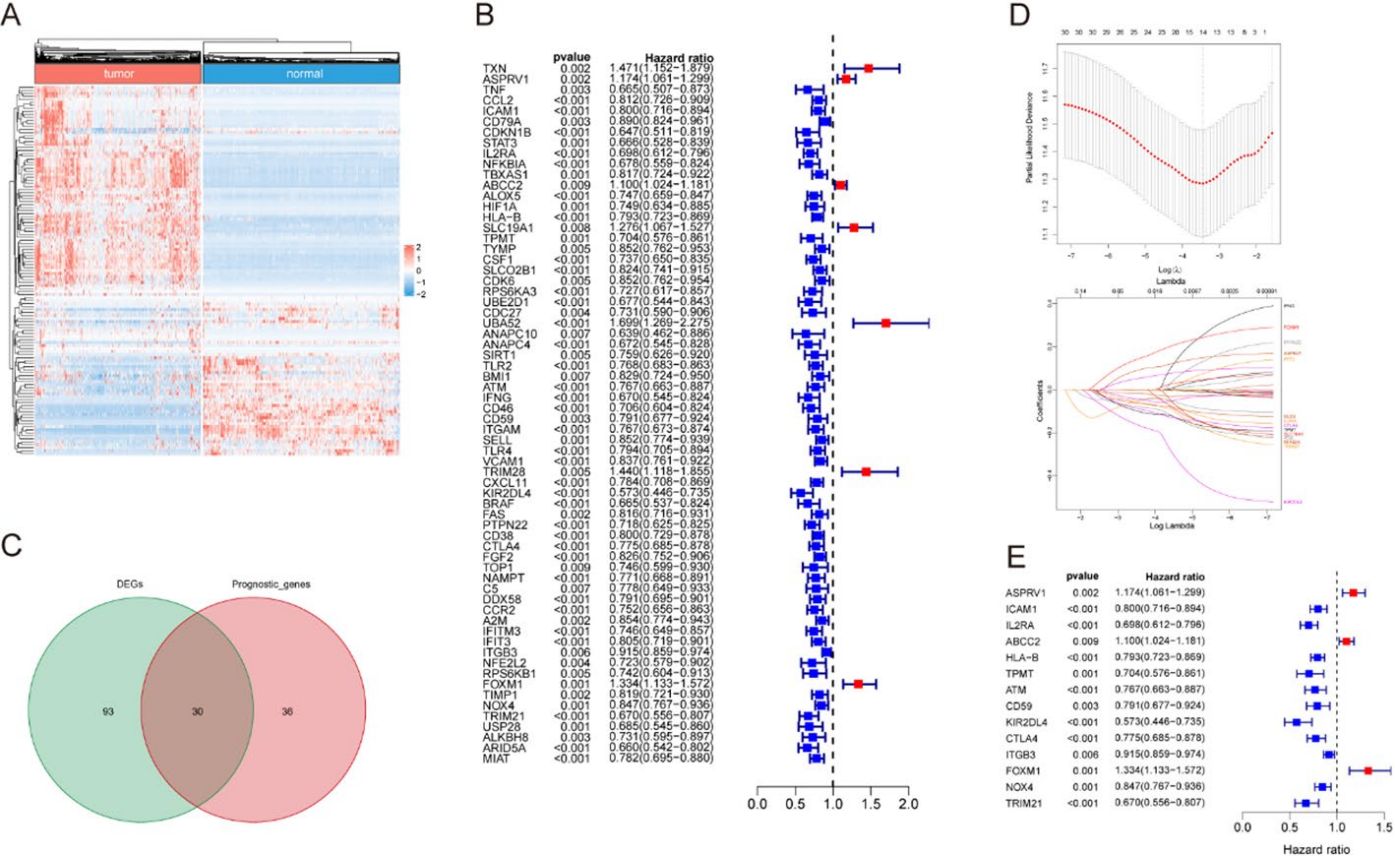
Identification and verification of differential expressed and prognostic SASP-related genes in melanoma. **A** Heatmap of DEG SASP genes. **B** The univariate Cox regression analysis revealed hazard ratios of 66 genes correlated with Melanoma prognosis (*p* < 0.01). **C** Venn diagram showing a total of 30 common SASP genes between the DEGs and prognosis genes. The LASSO regression analysis (**D**) and univariate Cox regression analysis (**E**) were employed to narrow the most relevant prognostic genes

RNA-seq data of 470 TCGA-SKCM samples and 555 GTEx normal skin samples were obtained from UCSC Xena. Among the 324 SASP-related genes picked up from GeneCards, 123 DEGs (48 downregulated and 75 upregulated) were identified in melanoma tissues as compared to normal skin samples with a criterion of FDR < 0.05 and | log2 (Fold Change) | > 1 (Fig. 1A). Meanwhile, 66 out of 324 SASP-related genes were significantly associated with the length of overall survival time in patients with melanoma (Fig. 1B). Subsequently, 30 candidate genes present in both of the lists of DEGs and prognosis-associated genes were selected for following model construction (Fig. 1C). Lasso regression was used to perform dimensionality reduction and a total of 14 genes including *ASPRV1*, *ICAM1*, *IL2RA*, *ABCC2*, *HLA-B*, *TPMT*, *ATM*, *CD59*, *KIR2DL4*, *CTLA4*, *ITGB3*, *FOXM1*, *NOX4*, and *TRIM21* were selected to calculate the risk score for patients with melanoma (Fig. 1D, E).

### Associations between risk score and survive outcome in patients with melanoma

**Fig. 2:**
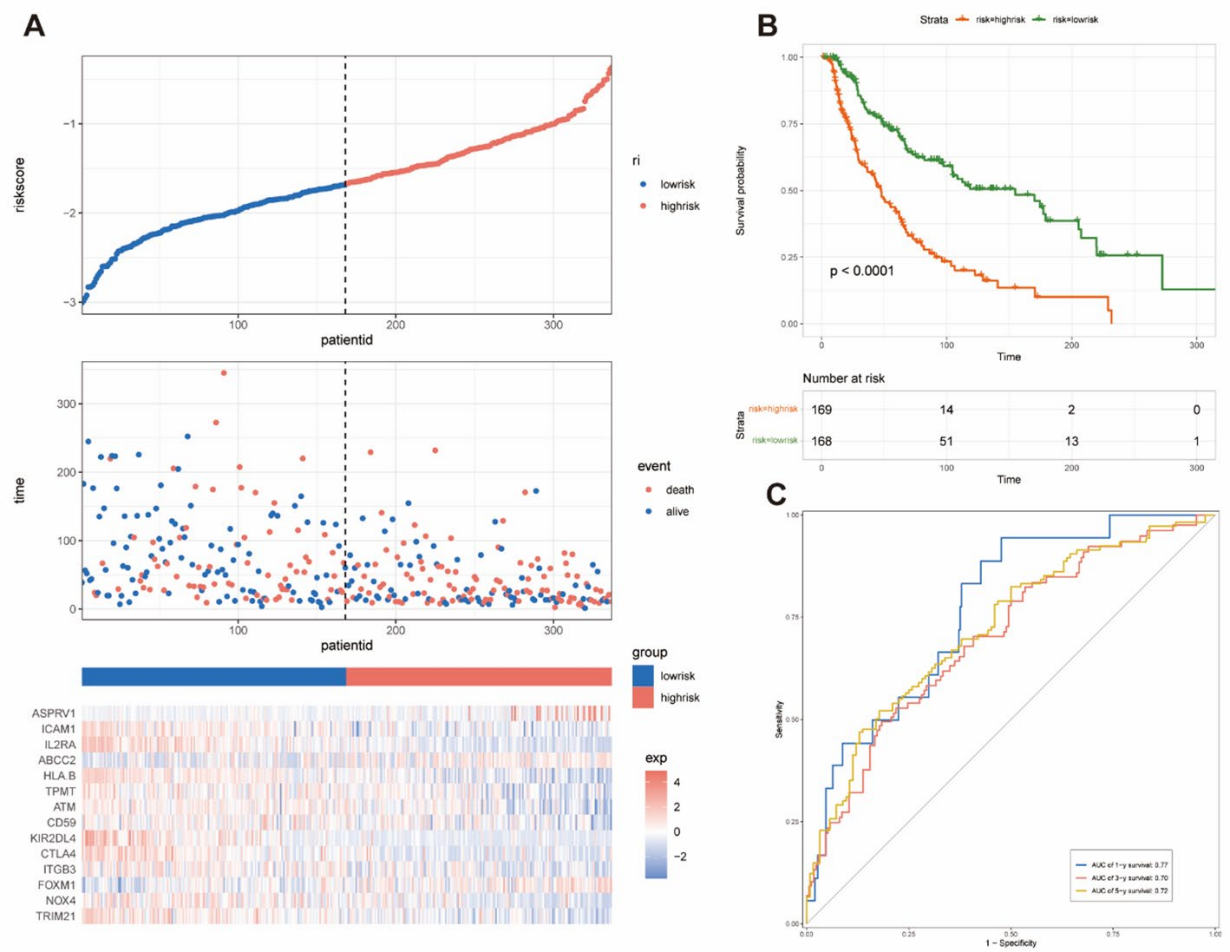
Prognostic analysis of the prognosis model in the TCGA-SKCM dataset. **A** The Kaplan-Meier analysis of the OS in the high-versus low-risk group. **B** The distribution of risk scores, survival status, and expression patterns of each melanoma patient in the TCGA dataset was visualized. **C** The ROC curve analyses showing the AUC values for the 1-, 3-, and 5-years survival rates were 0.77, 0.70, and 0.72, respectively

Patients from TCGA-SKCM cohort were divided into either high- or low-risk group according to risk scores (Fig. 2A). Worse overall survival outcome was observed in patients with high-risk score as compared to those with low-risk score (Fig. 2B). Moreover, the predicted AUC values of the 1-year, 3-years, and 5-years survival rates were 0.77, 0.70, and 0.72, respectively, indicating the satisfactory predictive ability of prognostic model (Fig. 2C).

**Fig. 3:**
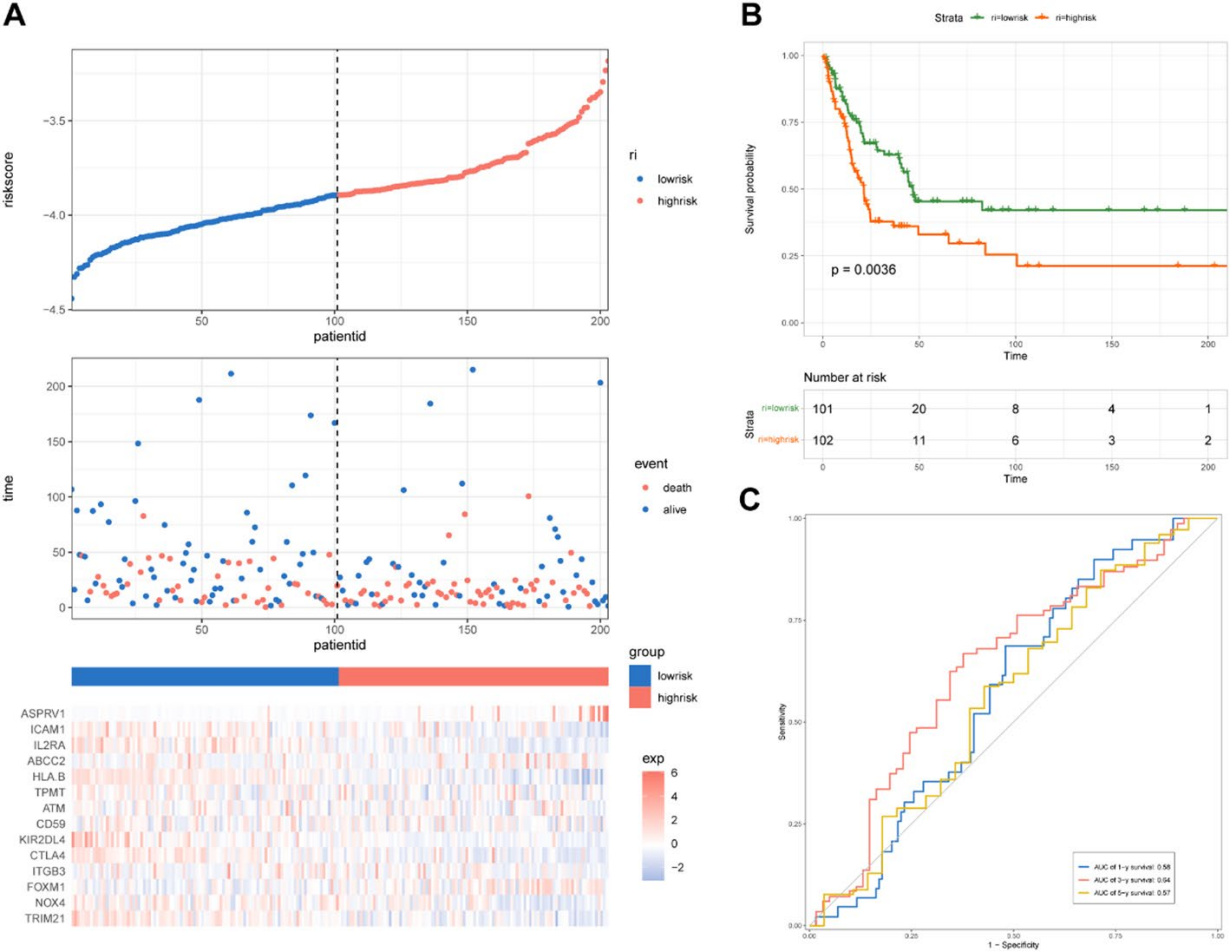
Prognostic analysis of the prognosis model in the GSE65904 dataset. **A** The Kaplan-Meier analysis of the OS in the high-versus low-risk group. **B** The distribution of risk scores, survival status, and expression patterns of each melanoma patient in the TCGA dataset was visualized. **C** The ROC curve analyses showing the AUC values for the 1-, 3-, and 5-years survival rates were 0.58, 0.64, and 0.57, respectively

To validate the stability and versatility of prognostic model, the risk scores were calculated for patients from GSE65904 dataset (Fig. 3A). Consistent with the TCGA-SKCM cohort, patients in high-risk group exhibited poor prognosis as compared to those in low-risk group (Fig. 3B). Furthermore, the predicted AUC values of the 1-year, 3-years, and 5-years survival rates were 0.58, 0.64, and 0.57, respectively (Fig. 3C).

### Establishment of prognostic nomogram for melanoma

**Fig. 4:**
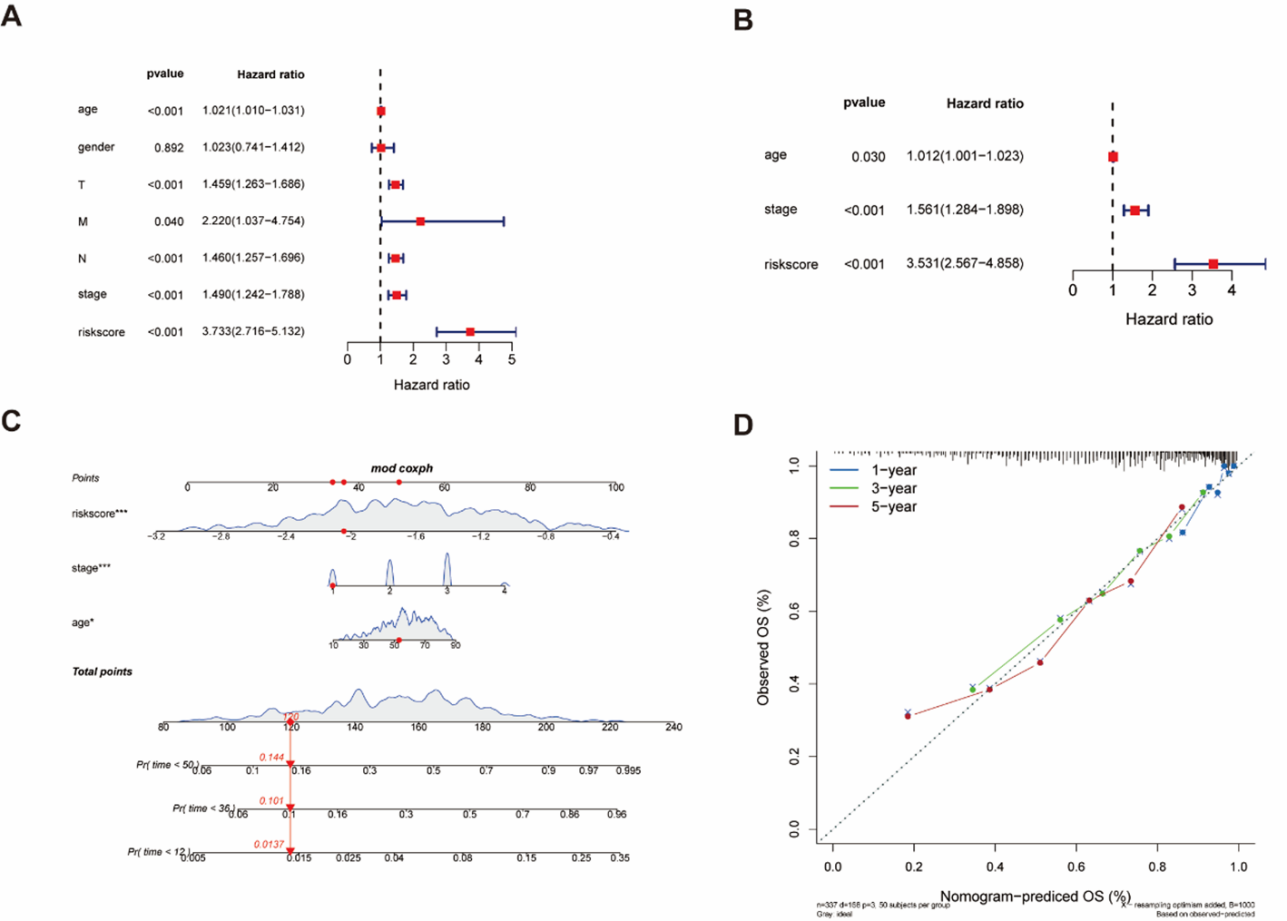
Univariate and multivariate cox analyses and the prognostic accuracy of risk score, age, stage, and TNM staging were compared. The univariate (**A**) and multivariate (**B**) Cox regression analyses of the risk score and other clinical feature prognostic values. **C** Based on all independent factors nomogram was constructed to predict the 1-, 3-, and 5-years OS of melanoma patients. **D** The calibration curve for the prediction and observed 1-, 3-, and 5-years OS

Univariate Cox analyses revealed that age (HR = 1.021, *P* < 0.001), T stage (HR = 1.459, *P* < 0.001), M stage (HR = 2.220, *P* = 0.040), N stage (HR = 1.460, *P* < 0.001), clinical stage (HR = 1.490, *P* < 0.001), and risk score (HR = 3.733, 95% CI = 2.716–5.132, *P* < 0.001) were significantly associated with OS in patients with melanoma (Fig. 4A). Moreover, multivariate Cox analysis demonstrated that risk score (HR = 3.531, *P* < 0.001), age (HR = 1.012, *P* = 0.030), and clinical stage (HR = 1.561, *P* < 0.001) were independent prognostic factors for melanoma (Fig. 4B). Based on these variables, a nomogram was developed to predict 1-, 3-, and 5-year OS for patients with melanoma (Fig. 4C). Calibration curve analysis showed that the predicted curves for 1-, 3-, and 5-year OS were in substantial agreement with the observed OS curves (Fig. 4D), indicating satisfactory accuracy and reliability of the nomogram.

### Functional characteristics of prognostic model

**Fig. 5:**
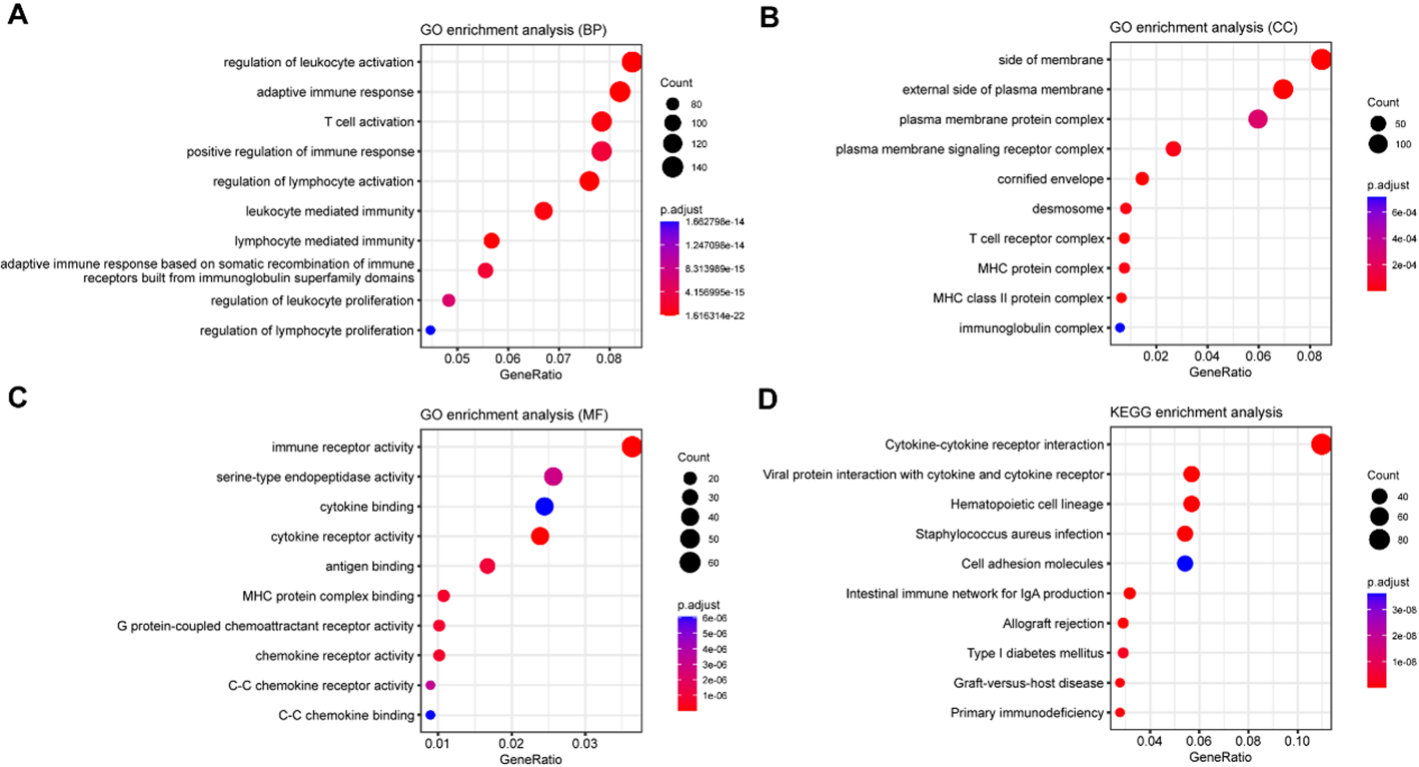
Analyses of functional enrichment. Bubble graph showing GO enrichment including BP, biological process (**A**); CC, cell component (**B**) MF, molecular function (**C**). **D** Bubble graph of KEGG enrichment (more dark red indicates more notable differences, and larger bubbles indicate more genes enriched)

To explore the underlying mechanism of the prognostic model based on SASP-related genes, we conducted differentially expression gene analysis between high- and low-risk groups using R package DESeq2 with a threshold of |log_2_FC| > 1 and *P* value < 0.01. As a result, 2369 DEGs (1344 up-regulated and 1025 down-regulated) were identified in the high-risk group as compared with low-risk group. The Gene Ontology (GO) enrichment analyses revealed that DEGs were significantly clustered in immune-related pathways, including regulation of leukocyte activation, adaptive immune response, T cell activation, positive regulation of immune response, and immune receptor activity (Fig. 5A–C). Furthermore, Kyoto Encyclopedia of Genes and Genomes (KEGG) pathway enrichment analysis showed significant enrichment of DEGs in pathways of cytokine-cytokine receptor interaction and viral protein interaction with cytokine and cytokine receptor (Fig. 5D). Collectively, these findings suggest that SASP-related genes may play a role in regulating the tumor microenvironment, thereby influencing the prognosis of melanoma.

### Characteristics of immune cells infiltration and immunotherapy prediction

**Fig. 6:**
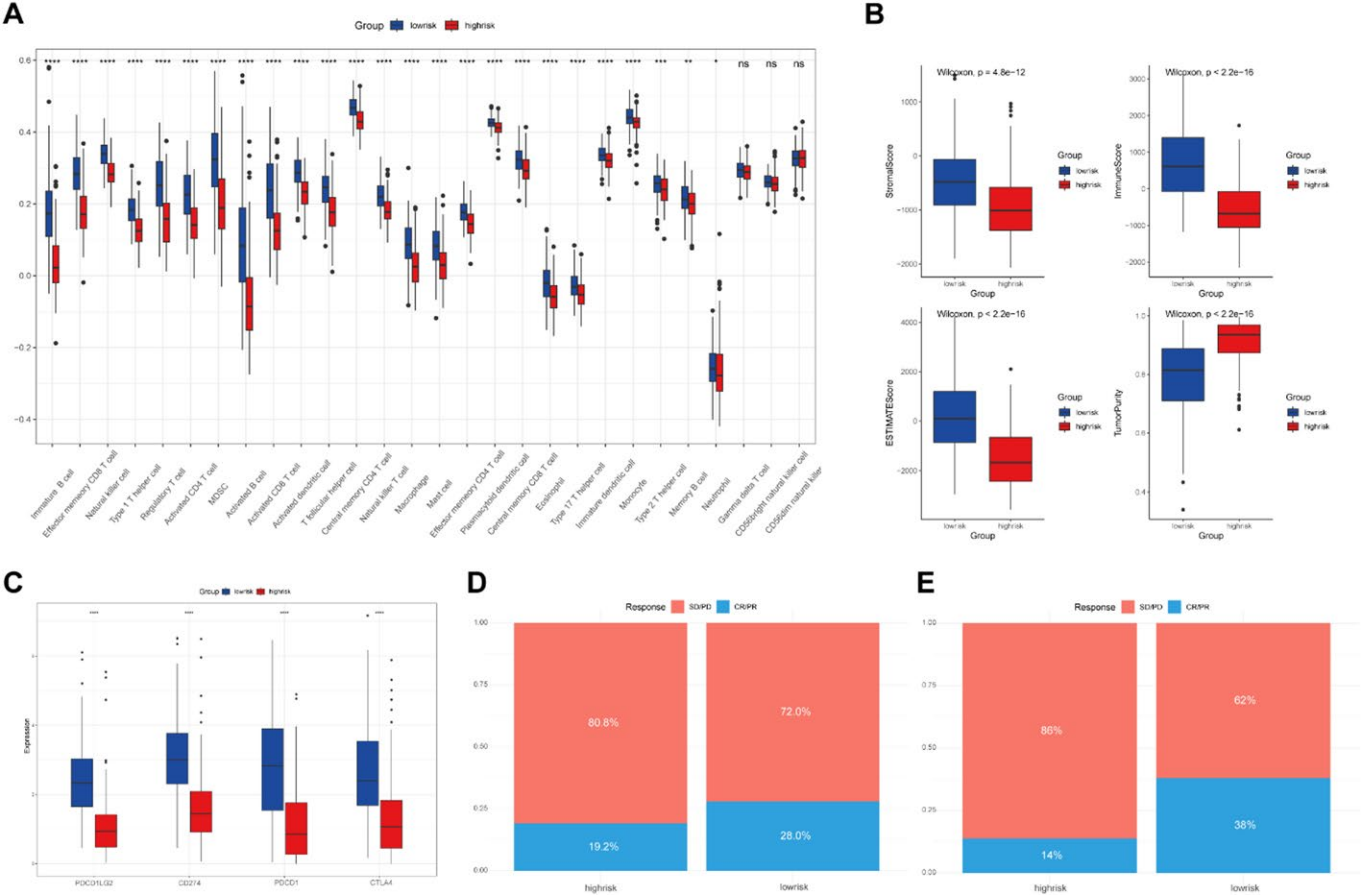
The prognosis model predicts immune status and the responsiveness to immunotherapy. **A** The ssGSEA algorithm showing the correlation between risk model and 28 immune cells. **B** RiskScore and ImmuneScore, StromalScore, ESTIMATEScore, and TumorPurity correlation analysis in TCGA dataset. **C** The expression of PD1, PD-L1, PD-L2 and CTLA-4 in the high-versus low- risk group. Rate of 109 melanoma samples’ clinical responses to pre- (**D**) or on **E** ICI treatment in high and low risk scores in GSE91061

To further investigate the association between SASP-related genes-based prognostic risk score and immune cell infiltration status in tumor immune microenvironment of melanoma, ssGSEA was applied to assess the proportion of infiltrated immune cells in TCGA-SKCM samples. Except for three subsets of tumor infiltrated immune cells including Gamma delta T cell, CD56 bright natural killer cell, and CD56 dim natural killer cell, the other 25 out of 28 subsets of tumor infiltrated immune cells showed a dramatic decrease in high-risk group as compared to low-risk group (Fig. 6A). Moreover, TME scores such as StromalScore, ImmuneScore, and ESTIMATEScore were significantly decreased whereas TumorPurity was elevated in high-risk group (Fig. 6B). Furthermore, the expression of CTLA-4, PD-1, PD-L1, and PD-L2 were substantially inhibited in high-risk group as compared with low-risk group (Fig. 6C). The above findings indicated the higher sensitivity of low-risk patients to ICI treatment. Ultimately, we evaluated the prediction of the risk score in a real immunotherapy cohort and found that low-risk patients at either pre- or on- ICI treatment state showed a higher relative probability of response to ICI (Fig. 6D, E).

## Discussion

Developing from transformed melanocytes, melanoma is the most invasive and deadly form of skin cancer [22]. Therefore, it is of great importance to identify the patients with increased progressive risk who could profit in early adjuvant therapies and the subsequent target or ICI treatments. However, the traditional clinical and histological indicators are insufficient to accurately identify these patients. In the present study, we constructed a prognostic risk-scoring model based on the expression of 14 SASP-related genes including *ICAM1*, *IL2RA*, *HLA-B*, *TPMT*, *ATM*, *CD59*, *KIR2DL4*, *CTLA4*, *ITGB3*, *NOX4*, *TRIM21*, *ASPRV1*, *ABCC2*, and *FOXM1*. In both of training cohort and validation cohort, patients with high-risk score suffered inferior prognosis. Moreover, the risk score was demonstrated to be an independent risk factor for prognosis in SKCM patients by multivariate Cox regression models. Furthermore, the nomogram was employed to quantitatively evaluate the 1-, 3-, and 5-year survival probability for melanoma patients.

Accumulating evidences have confirmed the critical role of senescence in tumor, but the role of senescence particularly SASP in the prognosis of melanoma is still not fully elucidated. Among the 14 genes for model construction, *ICAM1* is a key regulator of cellular responses in inflammation, injury resolution, and tumorigenesis [23]. A recent study found that blocking the expression of ICAM1 reduced both tumor size and metastasis of melanoma in older mice, suggesting ICAM1 could be a potential treatment target in elder patients with melanoma [24]. *IL2RA*, encoding the α chain of the IL-2 receptor complex, was found to be involved in immune homeostasis and cellular senescence [25]. Moreover, elevated expression of *IL2RA* might contribute to metastasis and unfavorable prognosis of melanoma via regulation of JAK-STAT signaling pathway and regulatory T-cells in the tumor microenvironment [26]. Furthermore, KIR2DL4 was found to be highly expressed in melanoma and contribute to the immunotherapy effect and prognosis in patients with melanoma via activating the immune cells such as NK cells and T cells [27]. The protein encoded by *CTLA4* is mainly expressed in tumor cells and a subset of tumor-infiltrating immune cells in melanoma. The pattern of DNA methylation change in the promoter regions of *CTLA4* is associated with the immunotherapy response and progression-free survival in patients with melanoma [28]. As a marker and important regulator of senescence, integrin subunit β3 (encoded by *ITGB3*) was previously reported to drive the distant metastasis of melanoma [29]. NOX4 is the most common isoform of NOX. Silencing of *NOX4* significantly inhibited basal ROS production and decreased melanoma cell viability through the FAK pathway [30]. ABCC2 (ATP Binding Cassette Subfamily C Member 2), also known as multidrug resistance-associated protein 2 (MRP2), is a protein encoded *ABCC2*. Over-expression of *ABCC2* in melanoma cells leads to resistance to chemotherapy drugs and unfavorable prognosis [31]. FOXM1 (Forkhead Box M1) is a transcription factor belonging to the FOX family subfamily. The high-level expression of FOXM1 has been validated in various cancer types including melanoma [32]. The evidences from previous studies have demonstrated the important role of these above-mentioned genes in the prognosis of melanoma and solidify the prognostic value of our risk-scoring model. Moreover, the rest of SASP-related genes recruited in our risk-scoring model including *HLA-B*, *TPMT*, *ATM*, *CD59*, *TRIM21*, and *ASPRV1* have not been well studied in melanoma, indicating their potential of novel therapeutic target.

Immune checkpoint inhibitor (ICI) treatment blocking PD-1, PD-L1, PD-L2, and CTLA4 has been demonstrated to be an effective treatment for various cancers including melanoma [33]. However, the variable response in patients with melanoma limited the precise treatment decisions and improvement of survival outcome in patients with melanoma [34]. Cytokine-induced senescence is an important mediator of cancer immune control underlying various immunotherapeutic approaches [35]. Moreover, treatment with SASP-associated cytokines supports the immune system’s self-sustained surveillance of senescent cells in melanoma [36]. In the present study, the DEGs between high-risk and low-risk groups were significantly enrich in immune-related pathways and cytokine-cytokine receptor interaction pathway by using GO enrichment and KEGG pathway enrichment analyses, respectively. Furthermore, the expression of PD-1, PD-L1, PD-L2, and CTLA-4 were observed to be overexpressed in the low-risk group. These findings suppose the potential use of our risk-scoring model in planning of ICI therapy strategy and predicting the immunotherapeutic efficiency.

In conclusion, we evaluated the prognostic signature and response to ICI in SKCM. Risk stratification based on the SASP-related prognostic signature was negatively related to clinical prognoses and levels of immune infiltration in patients. Furthermore, the model revealed that patients with low-risk scores were more likely to benefit from the ICI treatment. Our results could be beneficial in clarifying the role of SASP in the TME of SKCM. To summarize, the constructed prognostic signature could be applied clinically to increase survival as well as provide a novel target for treating SKCM patients in the future. Nonetheless, our study also has some limitations including lack of independent internal or external laboratorial validation of the newly developed prognostic model, as well as comparison with other existing prognostic markers/models, which is warranted in the future study.

## Data Availability

Data is provided within the manuscript or supplementary information files.
